# Early intervention for adolescents with Patellofemoral Pain Syndrome - a pragmatic cluster randomised controlled trial

**DOI:** 10.1186/1471-2474-13-9

**Published:** 2012-01-27

**Authors:** Michael S Rathleff, Ewa M Roos, Jens L Olesen, Sten Rasmussen

**Affiliations:** 1 Graduate School of Health Sciences, Aarhus University, Vennelyst Boulevard 9, 8000 Aarhus C, Denmark; 2 Orthopaedic Surgery Research Unit, Aalborg Hospital - Aarhus University Hospital, Soendre Skov 15, 9000 Aalborg, Denmark; 3 Research Unit for Musculoskeletal Function and Physiotherapy, Institute of Sports Science and Clinical Biomechanics, University of Southern Denmark, Campusvej 55, 5230 Odense M, Denmark; 4 Department of Rheumatology, Aalborg Hospital - Aarhus University Hospital, Reberbansgade 15, 9000 Aalborg, Denmark

**Keywords:** Patellofemoral Pain Syndrome, Anterior Knee Pain, Physiotherapy, Adolescents

## Abstract

**Background:**

Self-reported knee pain is highly prevalent among adolescents. As much as 50% of the non-specific knee pain may be attributed to Patellofemoral Pain Syndrome (PFPS). In the short term, exercise therapy appears to have a better effect than patient education consisting of written information and general advice on exercise or compared with placebo treatment. But the long-term effect of exercise therapy compared with patient education is conflicting. The purpose of this study is to examine the short- and long-term effectiveness of patient education compared with patient education and multimodal physiotherapy applied at a very early stage of the condition among adolescents.

**Methods/Design:**

This study is a single blind pragmatic cluster randomised controlled trial. Four upper secondary schools have been invited to participate in the study (approximately 2500 students, aged 15-19 years). Students are asked to answer an online questionnaire regarding musculoskeletal pain. The students who report knee pain are contacted by telephone and offered a clinical examination by a rheumatologist. Subjects who fit the inclusion criteria and are diagnosed with PFPS are invited to participate in the study. A minimum of 102 students with PFPS are then cluster-randomised into two intervention groups based on which school they attend. Both intervention groups receive written information and education. In addition to patient education, one group receives multimodal physiotherapy consisting primarily of neuromuscular training of the muscles around the foot, knee and hip and home exercises.

The students with PFPS fill out self-reported questionnaires at baseline, 3, 6, 12 and 24 months after inclusion in the study. The primary outcome measure is perception of recovery measured on a 7-point Likert scale ranging from "completely recovered" to "worse than ever" at 12 months.

**Discussion:**

This study is designed to investigate the effectiveness of patient education compared with patient education combined with multimodal physiotherapy. If patient education and multimodal physiotherapy applied at an early stage of Patellofemoral Pain Syndrome proves effective, it may serve as a basis for optimising the clinical pathway for those suffering from the condition, where specific emphasis can be placed on early diagnosis and early treatment.

**Trial Registration:**

clinicaltrials.gov reference: NCT01438762

## Bagground

Self-reported knee pain is highly prevalent among adolescents [1,2]. Cross-sectional studies show that between 18.5 and 31% of adolescents report knee pain[1,3]. As much as 50% of the non-specific knee pain may be attributed to Patellofemoral Pain Syndrome (PFPS) [4]. The most frequently reported symptoms in PFPS are a diffuse peripatellar and retropatellar localised pain, typically provoked by ascending or descending stairs, squatting, cycling and sitting with flexed knees for prolonged periods of time [5].

The prevalence of PFPS has been reported across several age groups, with females having a prevalence approximately 1.5-3 times higher than males in athletic populations [6,7]. Among patients ranging from 10 to 49 years of age reporting to a sports medicine clinic, 70% of those diagnosed with PFPS were between the ages of 16 and 25 years [8]. The high prevalence of PFPS among young people is further supported by a recent study that examined 299 students aged 16-18 years [4]. They found that 25% of the students had experienced knee pain during the previous month. Of the students with knee pain, 50% were diagnosed with PFPS. Only two studies have investigated the incidence of PFPS within a closed population. Witvrouw et al (2000) followed 282 students of physical education and found that 9% developed PFPS during a two-year period [9]. Boling et al followed 1597 midshipmen for up to 2.5 years and found a significantly lower incidence of 3% [10].

There are a number of different treatment options for PFPS. Exercise therapy has been advocated as one of the cornerstones in rehabilitation of patients with PFPS. In the short term, exercise therapy appears to have a better effect than patient education consisting of written information and general advice on exercise [11] or compared with placebo treatment [12]. But the long-term effect of exercise therapy compared with patient education or flat insoles is conflicting. After 12 months of treatment, Collins et al. found no difference in pain or function between patients receiving multimodal physiotherapy (consisting of patellofemoral joint mobilisation, patellar taping, quadriceps muscle retraining, and education) or flat insoles [13]. Contrary to these results, van Linschoten et al. found a significant effect of supervised exercise therapy compared with patient education on self-reported pain levels after 12 months, but there was no positive effect on self-reported function or self-perceived recovery [11].

One possibility to improve short- and long-term outcomes is to initiate treatment early in the clinical course. Predictors of long-term outcome (> 52 weeks) indicate that a long symptom duration [14,15], higher age [16] and greater pain severity at baseline [14] are associated with poorer outcome. These prognostic factors suggest that an early initiation of treatment among adolescents may lead to a better long-term prognosis. The purpose of this study is to examine the short- and long-term effectiveness of patient education compared with patient education and multimodal physiotherapy applied at a very early stage of the condition among adolescents. We hypothesise a significantly larger proportion of completely recovered students at the 12 months follow-up in the patient education combined with multimodal physiotherapy treatment option compared with patient education alone.

## Methods

### Design

The study involves a single blind pragmatic cluster randomised controlled trial. The protocol conforms to CONSORT guidelines for non-pharmacological interventions [17] and is approved by the local ethics committee in North Denmark Region (N-20110020). All students below 18 years of age are required to give informed together with parental consent. Students aged 18 or 19 are allowed to give informed consent without parental consent.

### Patient selection

**Figure 1:**
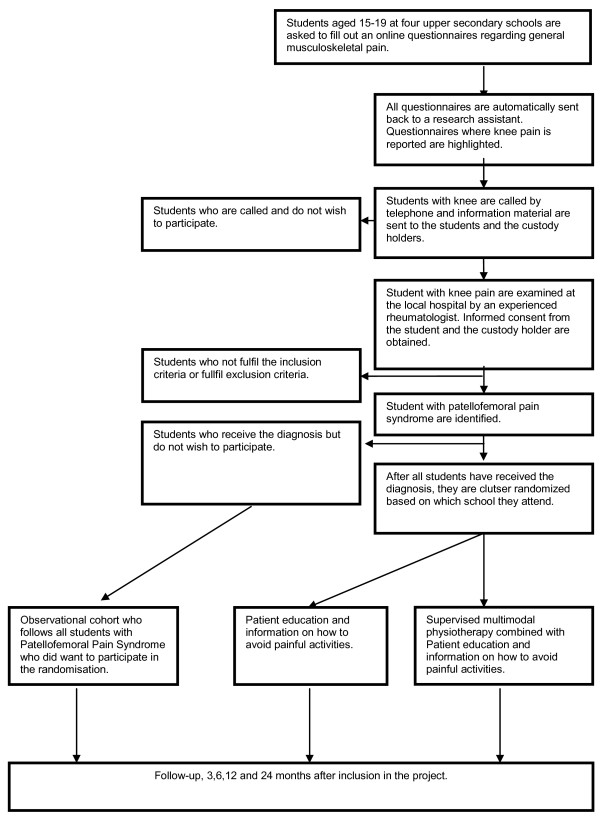
**Flowchart**. Subjects who are diagnosed with PFPS will be invited to participate in the study. 104 students diagnosed with PFPS will be cluster randomised into two groups based on which school they attend.

Four upper secondary schools were invited to participate in the study. All four schools accepted the invitation. All students (approximately 2500), aged 15-19 years, in the schools are invited to answer an online questionnaire as part of their physical education lessons. The students who report knee pain are contacted by telephone and offered a clinical examination by an experienced rheumatologist who evaluates which students are eligible to enter the study (Figure 1).

### Questionnaire

The online questionnaire contains questions regarding knee pain and general musculoskeletal pain, activity level, participation in sport, and quality of life measured by the EuroQol questionnaire (EQ5D). An EQ-5D score for all students will be calculated by using the official Danish time trade-off scores[18].

Localisation of pain is measured with a Pain Mannequin representing the entire body including the head. Students are asked if they have current pain in one or more of these areas. If they mark an area on the pain mannequin, they are asked how often they have felt pain in the area. The answer is divided into the following categories: rarely; monthly; weekly; more than one time per week; almost daily.

The physical education teachers will instruct the student in how to answer the questionnaire during a four-week period, starting two weeks after students have returned from summer holidays in 2011. After the questionnaires are filled out, they are automatically transferred to a secure server.

### Inclusion and exclusion criteria

Eligibility criteria are in line with a previous clinical trial [19]; age 15-19 years; insidious onset of anterior knee or retropatellar pain of greater than six weeks' duration and provoked by at least two of the following situations: prolonged sitting or kneeling, squatting, running, hopping, or stair walking; tenderness on palpation of the patella, or pain with stepping down or double leg squatting; and worst pain over the previous week of at least 30 mm on a 100 mm visual analogue scale (VAS). Exclusion criteria were concomitant injury or pain from the hip, lumbar spine, or other knee structures; previous knee surgery; patellofemoral instability; knee joint effusion; use of physiotherapy for treating knee pain within the previous year; or weekly use of anti-inflammatory drugs.

### Randomisation

Subjects who fit the inclusion criteria are offered to participate in the study. After all subjects have undergone a clinical examination and signed an informed consent, students are cluster-randomised into two intervention groups based on which school they attend. Cluster randomisation is chosen to minimise the bias that could be introduced if more than one student in each class is diagnosed with PFPS, but randomised to different treatment groups.

### Interventions

Both intervention groups receive written information and education delivered during one-to-one sessions with a physiotherapist.

### Information and education

One physiotherapist will carry out all patient education in the two clusters that are randomised to patient education only. The physiotherapist has previous experience in treating adolescents and PFPS and has more than two years of practical experience. The information and education is standardised and covers the topics of: why does it hurt: pain management; information on how to modify physical activity; how to return slowly to sports; how to cope with knee pain, and information on how to increase knee alignment during sit-to-stand, standing, walking, stair walking and bicycling. The patients will also receive this information in an eight-page leaflet. This session is expected to take approximately 30 minutes depending on the number of questions from the student or the parents.

### Information and education plus multimodal physiotherapy

The multimodal physiotherapy is carried out by two physiotherapists (one in each of the two clusters). Both have previous experience in treating adolescents and subjects with PFPS, and have more than two years of practical experience. The multimodal physiotherapy consists of patellofemoral soft tissue mobilisation, stretching of the muscles around the hip and knee, patellar taping, neuromuscular training of the muscles around the foot, knee and hip, quadriceps strength training for the knee and hip and instructions on home exercises [12,20]. The intervention is carried out on the school premises three times per week immediately after the end of class for a total of three months. Students are expected to vary greatly with regard to pain severity and level of function. To tailor the exercise therapy to the specific performance level of the student, all exercises are available in three or four different levels to enable progression (see Table 1). All students start with exercises at level one. Three general rules have been made to determine when progression of the exercises will occur:

**Table 1:** the exact exercise, number of repetitions and external load will be determined by the physiotherapist.

Functional retraining exercises performed three times a week	•Sitting (isometric) • Sit-to-stand • Single step up • Stair walking • Single leg squat

Quadriceps muscle strengthening performed three times/week	• Inner range (open kinetic chain) • Mid range (open kinetic chain) • Weight-bearing

Hip abduction strengthening performed three times a week	• Side-lying hip abduction • Side-lying hip abduction with rubber band for resistance • Standing, hip abduction of the non-weight bearing leg • Standing, hip abduction of the non-weight bearing leg with rubber band between the ankles as extra resistance • Side-lying bridge • Side-bridge with hip abduction

Stretching	• Hamstring muscle stretches in sitting • Anterior hip structures stretch. Subjects in prone position, one hip externally rotated and with both the hip and knee flexed • Patellofemoral joint mobilisation and soft-tissue will be performed by the physiotherapist and taught so patient can perform this themselves

Patellar taping	• Combination of tilt, medial glide and fat pad unloading as necessary- the tape will be applied by the physiotherapist each training session

(1) Good quality of movement determined by the physiotherapist. 'Good quality' is defined as able to control hip, knee and foot alignment during exercises with both extra-slow and slightly faster than normal movement.

(2) Ability to perform the actual number of repetitions as defined in the training protocol.

(3) No self-reported increase in usual pain after the training session or the next morning.

To improve compliance, students are offered the opportunity to attend the supervised group training session at either 12:45, 13:45. or 15:15 to account for variation in the time the school lessons end. The training sessions are available Mondays, Wednesdays and Fridays.

### Patellar taping

Patellar taping based on the McConnell approach is included as it may reduce pain during exercise[21]. Non-rigid, hypoallergenic tape (Curafix H, Lohmann & Rauscher, Neuweid Garmany) will be used to reduce skin irritation while rigid zinc-oxide tape (Leuko P, BSN Medical, Hamburg, Germany) is used for the corrections of the patella. Taping corrections are applied in a predetermined order of anterior tilt, medial tilt, glide, and fat pad unloading until the participant**'**s pain is reduced by at least 50% [12]. Tape is only used if patients achieve a minimum of 50% reduction in pain measured with VAS during a two-leg squat immediately after application of the tape. Students are taught to independently apply the taping corrections and are instructed to reapply the tape daily and wear the tape during waking hours for the duration of the trial.

### Home exercises

Home exercises consist of quadriceps and hip muscle retraining and stretching. The exercises are performed each day in accordance with the regime proposed by McConnell [12].

### Compliance

Compliance with the supervised intervention is recorded as the number of times each student participates in the group training. Good compliance is defined as participation in at least 80% of the supervised group training sessions. Poor compliance is defined as participation in less than 40% of the group training sessions. Compliance in home exercises will be monitored by weekly follow-up using Short Message System (SMS). Good compliance in home training is defined as self-reported participation in at least 70% and poor compliance is defined as below 40%.

### Adherence to treatment

To increase adherence to the designated intervention and to optimise retention, parents are invited to participate in all aspects of the study. Communication with the students is done by telephone or email. The day before appointments, students are sent a reminder by SMS. If students know in advance that they cannot participate in the group training sessions, they are asked to send an SMS to the physiotherapist that states they will not be participating.

If students do not show up for training twice in a row, and do not cancel through SMS they are telephoned by the physiotherapist who asks them in a friendly manner when they will return. If the students do not answer the phone twice or do not call back, they receive an SMS that tells them that they will be contacted the next day. If they fail to reply to the SMS or do not answer the phone the following day, they are sent a letter requesting contact.

### Co-interventions

Students with PFPS are asked to refrain from all other co-interventions during the intervention period starting 72 hours before participation in the study. Pre-existing foot orthoses are allowed, but patients are not allowed to change orthoses or modify their current orthoses during the study period. Current or prior analgaesic use for the current knee pain is registered during baseline testing and all follow-ups.

### Observational cohort

Those who do not wish to participate in the randomisation procedure are followed as an observational cohort. The observational cohort is followed at the same time-points and is asked which treatment they have received.

### Outcome measurements

#### Self-reported outcome measurements

Self-reported questionnaires are filled out by the students with PFPS at baseline, 3, 6, 12 and 24 months after inclusion in the study. A physiotherapist not involved in the treatment, will hand out all questionnaires during follow-up and answer questions from the students. The primary outcome measure is perception of recovery after 12 months measured on a 7-point Likert scale ranging from "completely recovered" to "worse than ever". Students are categorised as "recovered" if they rate themselves as **"**fully recovered**" **or **"**strongly recovered**"**. Students rating themselves in one of the other five categories from **"**slightly recovered**" **to **"**worse than ever" are categorised as "not recovered" [11]. Secondary outcomes include Knee Injury and Osteoarthritis Outcome Score (KOOS) [22]. The change from baseline to each point of follow-up in the average score of the five KOOS subscale scores (KOOS_5_) covering pain, symptoms, activities of daily living, difficulty in sports and recreational activities, and quality of life are used. Further, EQ5D is used as s self-reported generic measure of health status [23].

#### Objective outcomes

A subgroup of 15 female students with PFPS from each cluster (a total of 60 students) is invited to participate in a sub-study investigating the effect of interventions on changes in neuromuscular function and isometric strength of the quadriceps. A person not involved in the treatment or diagnostics of the students randomly calls newly diagnosed subjects and asks them to participate. The students will undergo quadriceps strength measurements and two tests of neuromuscular function of m. vastus medialis and m. vastus lateralis before treatment and again after three months of treatment. Surface Electromyography (sEMG) is collected during two different conditions: stair walking and semi-squat at 90 degrees flexion at the knee joint. These measurements are carried out with the assessor blinded and before students are randomised. At three months follow-up, the same assessor tests all 60 students again. Before students are tested at three months follow-up, they are told not to reveal which intervention group they were assigned to. The assessor does not participate in any other parts of the study.

Bipolar surface electrodes (Ambu A/S, Neuroline, Ballerup, Denmark) are placed on the muscle bellies of m. vastus medialis (VM) and lateralis (VL) with an interelectrode distance of 22 mm. The electrode for VM is placed over the muscle belly 4 cm superior to and 3 cm medial to the superomedial patella border and orientated 55° to the vertical. The electrode for VL is placed 10 cm superior to and 6-8 cm lateral to the superior border of the patella and orientated 15° to the vertical [24]. The ground electrode is placed over the tibial tubercle.

#### Isometric strength measurement

The testing set up includes a portable handheld dynamometer (HHD) and an examination table. Muscle strength is tested with the Power track II commander (JTECH Medical, Salt Lake City, Utah, USA). A physiotherapist with previous experience using the HHD performs all measurements. Only isometric extension strength of the knee is tested.

The test position is chosen based on procedures that are often applied in clinical settings and have proven reliable[25]. The students are told to stabilise themselves by holding onto both sides of the table with their hands. Using a secure strap tied to the examination table the dynamometer is secured to the table and the patient exerts a 5-sec. isometric maximum voluntary contraction (MVC) against the dynamometer. Students are asked to perform one isometric sub-maximal contraction, to ensure that the correct action is performed. After a 30-sec. rest period the individual test is performed four times. The highest value of four consecutive measurements and the mean of the three highest values are used as the outcome. Students are given a 30-sec. rest period between each trial. The standardised command by the examiner is ''go ahead-push-push-push-push and relax''.

### Semi squat

After isometric strength measurement, sEMG is recorded during an isometric holding test. The holding test consists of a semi-squat. Students are asked to position themselves with a 90-degree knee angle, and to keep as steady as possible. They perform one practice trial of 5 sec. After the practice trial they have 2 minutes of rest. Afterwards they are asked to resume the position and when the knees are in a 90-degree angle, the sEMG recordings of VM and VL and signal from the knee goniometer start. A total of 20 consecutive seconds is recorded. The sEMG-signal from VM and VL are analysed with respect to quantifying the nonlinear complexity of the sEMG signal as a composite measure of neuromuscular control [26].

### Stair walking

Students walk up and down a stairway with 10 consecutive steps. They walk up and down once corresponding to 10 steps ascending and 10 steps descending. An electronic knee goniometer is used to record the flexion/extension movement at the knee. The knee goniometer consists of a potentiometer attached to two thermoplastic cups that secure around the tibia and femur. sEMG from VM and VL and kinematics are recorded from the most painful knee.

### Sample size

From the study done by Moelgaard et al [4], we expect that at least 6% of the students will be diagnosed with PFPS. This corresponds to at least 150 students with PFPS among the 2500 students.

Based on the study done by van Linschoten et al [11] and Clark et al [27] we expect a 30% difference between intervention groups in the two categories "recovered" and "not recovered". We expect a 20% recovery in the patient education group, and 50% recovery in the group receiving patient education combined with multimodal physiotherapy. Sample size calculations show that at least 51 subjects in each group are needed to detect a statistical difference (power 0.90, alpha 0.05). To account for a drop-out rate of 10%, a minimum of 56 patients is included in each group.

The sample-size of 60 students (2 × 30 students) in the sub-study is based on the complexity of the sEMG-signal from VM during semi-squatting. Based on pilot data, 25 students are needed in each group to detect a 15% difference (0.104 vs. 0.120 Sample Entropy, standard deviation of 0.02, 80% power) between groups at three-months follow-up. To account for loss to follow-up, we increase the sample size to 2 × 30.

### Statistical analysis

All RCT analyses take place after the 12 months follow-up and no intermediate analyses are performed. Publication of the results from the trial will take place after the two-year follow-up. Between-group comparison is analysed on an intention-to-treat basis. Comparison of the primary dichotomous outcome is analysed through logistic regression for repeated measurement coded as "recovered" or "not recovered". Logistic regression is adjusted for baseline values and prognostic factors such as gender and duration of symptoms. These adjustments are only included in the final model if the estimate changes more than 10% when entering the variables in the model. Secondary analysis includes a per-protocol analysis and a predefined subgroup analysis investigating the interaction between treatment and compliance.

### Data analysis of sEMG recordings

Sample entropy (SaEn) is used to quantify the complexity of the time series from the EMG recordings during semi-squatting. Entropy quantifies the complexity of a dataset by assessing the probability that equal sequences of length **m **remain similar after a time increment. The degree of similarity is determined by the tolerance **r**. The output is a unitless, non-negative number where lower values indicate a more regular signal and higher values a more complex signal. The sEMG signal is divided into 5 cycles consisting of 4 sec each to account for possible time-dependent changes in the sEMG-signal. For more information about computation see [28].

The automatic algorithm used to identify the onset of VM and VL during stair walking identifies the point where the sEMG-signal deviates more than three standard deviations, for a minimum of 25 ms from the baseline level taken 75 ms before the foot touches the stair [29]. The sEMG onset is reported as an average taken over 10 steps. The relative difference in the onset of EMG activity of VM and VL is calculated by subtracting the onset of VM from that of VL. This method has been used earlier and shows excellent reliability (ICC > 0.90) [29].

## Discussion

There is a paucity of studies targeting prognostic factors for long-term outcome in patients with PFPS. Baseline variables such as a long symptom duration[14,15], higher age[16] and greater pain severity at baseline[14] are associated with a poorer outcome. These baseline variables suggest that early diagnosis and targeted treatment early in the clinical course could improve long-term prognosis.

### Strengths

There are several strengths of the recruitment and intervention design in this study. Firstly, recruitment through the upper secondary schools will enable us to include patients with shorter symptom duration, minor pain severity and younger age, than if recruitment were to take place through the general practitioner (GP).

The multimodal physiotherapy resembles the current clinical physiotherapy practice in Denmark and other countries [11,30]. The semi-structured nature of the exercise therapy incorporates a set of guidelines to help the physiotherapists determine when to reduce and when to progress the exercises and will help to reduce treatment variation and allow for easy reporting and implementation.

We have included three secondary functional outcomes that include strength measurement of the quadriceps, and two basic tests of neuromuscular control. Studies indicate that patients with PFPS have an altered neuromuscular function with delayed timing of the VM and VL [31] and a lower muscular strength of the quadriceps [32,33]. The goal of treatment is to restore normal function and reduce pain. These strength and neuromuscular measures may allow us to investigate the underlying mechanisms that may explain changes in pain and function among students with PFPS.

### Limitations

Studies often recruit patients through general practitioners (GPs) or sports clinics. This approach benefits from resembling the current practice but may delay the referral to physiotherapy as the 'wait and see' approach combined with patient education is one of the treatment options currently used in the primary sector in Denmark. The recruitment procedure used in the current study may decrease the external validity of the results as students are recruited earlier than if recruitment were to take place through sports clinics or GPs. However if the early intervention proves successful, it may serve as a basis for optimising the clinical pathway for patients with PFPS with a specific emphasis on early diagnosis and quick referral to physiotherapy.

One of the most frequently used outcome measures in patients with Patellofemoral Pain Syndrome is the Kujala Patellofemoral Score (KPS) [11,19,34]. The KPS has previously been used in assessing treatment outcome in patients with PFPS. The KPS is not available in the Danish language, which is why we choose to use a 7-point Likert scale ranging from "completely recovered" to "worse than ever". This scale has previously been used by van Linschoten et al [11] in a study resembling this study. As a secondary outcome, we will use the knee-specific patient-related outcome measure KOOS which has proven reliable, valid and has an excellent responsiveness [35].

## Conclusion

This study uses a pragmatic cluster randomised controlled design to investigate the effectiveness of patient education compared with patient education combined with multimodal physiotherapy. The recruitment procedure may decrease the external validity for patients seen in primary care as students are recruited through schools instead of through GPs. If patient education and multimodal physiotherapy applied at an early stage of Patellofemoral Pain Syndrome proves effective, it may serve as a basis for optimising the clinical pathway for those suffering from the condition, where specific emphasis can be placed on early diagnosis and early treatment.

## Competing interests

The authors declare that they have no competing interests.

## Authors' contributions

All authors made substantial scientific contributions to the design of the trial. MSR wrote the first draft for this manuscript. EMR, JLO and SR all made valuable scientific additions to the draft. All authors read and approved the final manuscript.

## Pre-publication history

The pre-publication history for this paper can be accessed here:

http://www.biomedcentral.com/1471-2474/13/9/prepub

## References

[B1] Fairbank JC Pynsent PB van Poortvliet JA Phillips H Mechanical factors in the incidence of knee pain in adolescents and young adults JBone Joint SurgBr 1984 66 5 685 693 10.1302/0301-620X.66B5.6501361 6501361

[B2] Stovitz SD Pardee PE Vazquez G Duval S Schwimmer JB Musculoskeletal pain in obese children and adolescents Acta Paediatr 2008 97 4 489 493 10.1111/j.1651-2227.2008.00724.x 18363957

[B3] Vahasarja V Prevalence of chronic knee pain in children and adolescents in northern Finland Acta Paediatr 1995 84 7 803 805 10.1111/j.1651-2227.1995.tb13760.x 7549301

[B4] Molgaard C Rathleff MS Simonsen O Patellofemoral Pain Syndrome and Its Association with Hip, Ankle, and Foot Function in 16- to 18-Year-Old High School Students: A Single-blind Case-control Study J Am Podiatr Med Assoc 2011 101 3 215 222 21622633 10.7547/1010215

[B5] Haim A Yaniv M Dekel S Amir H Patellofemoral pain syndrome: validity of clinical and radiological features Clin Orthop Relat Res 2006 451 223 228 16788411 10.1097/01.blo.0000229284.45485.6c

[B6] Taunton JE Ryan MB Clement DB McKenzie DC Lloyd-Smith DR Zumbo BD A retrospective case-control analysis of 2002 running injuries Br J Sports Med 2002 36 2 95 101 10.1136/bjsm.36.2.95 11916889 PMC1724490

[B7] DeHaven KE Lintner DM Athletic injuries: comparison by age, sport, and gender The American journal of sports medicine 1986 14 3 218 224 10.1177/036354658601400307 3752362

[B8] Devereaux MD Lachmann SM Patello-femoral arthralgia in athletes attending a Sports Injury Clinic BrJSports Med 1984 18 1 18 21 10.1136/bjsm.18.1.18 PMC1858870 6722419

[B9] Witvrouw E Lysens R Bellemans J Cambier D Vanderstraeten G Intrinsic risk factors for the development of anterior knee pain in an athletic population. A two-year prospective study The American journal of sports medicine 2000 28 4 480 489 10921638 10.1177/03635465000280040701

[B10] Boling MC Padua DA Marshall SW Guskiewicz K Pyne S Beutler A A prospective investigation of biomechanical risk factors for patellofemoral pain syndrome: the Joint Undertaking to Monitor and Prevent ACL Injury (JUMP-ACL) cohort The American journal of sports medicine 2009 37 11 2108 2116 10.1177/0363546509337934 19797162 PMC2860575

[B11] van Linschoten R van Middelkoop M Berger MY Heintjes EM Verhaar JA Willemsen SP Koes BW Bierma-Zeinstra SM Supervised exercise therapy versus usual care for patellofemoral pain syndrome: an open label randomised controlled trial BMJ 2009 339 b4074 10.1136/bmj.b4074 19843565 PMC2764849

[B12] Crossley K Bennell K Green S Cowan S McConnell J Physical therapy for patellofemoral pain - A randomized, double-blinded, placebo-controlled trial Am J Sport Med 2002 30 6 857 865 10.1177/03635465020300061701 12435653

[B13] Collins N Crossley K Beller E Darnell R McPoil T Vicenzino B Foot orthoses and physiotherapy in the treatment of patellofemoral pain syndrome: randomised clinical trial BMJ 2008 337 a1735 18952682 10.1136/bmj.a1735 PMC2572211

[B14] Collins NJ Crossley KM Darnell R Vicenzino B Predictors of short and long term outcome in patellofemoral pain syndrome: a prospective longitudinal study Bmc Musculoskel Dis 2010 11 11 10.1186/1471-2474-11-11 PMC2823664 20082723

[B15] Blond L Hansen L Patellofemoral pain syndrome in athletes: a 5.7-year retrospective follow-up study of 250 athletes Acta Orthop Belg 1998 64 4 393 400 9922542

[B16] Kannus P Niittymaki S Which factors predict outcome in the nonoperative treatment of patellofemoral pain syndrome? A prospective follow-up study Med Sci Sport Exer 1994 26 3 289 296 8183092

[B17] Boutron I Moher D Altman DG Schulz KF Ravaud P Extending the CONSORT statement to randomized trials of nonpharmacologic treatment: explanation and elaboration Ann Intern Med 2008 148 4 295 309 18283207 10.7326/0003-4819-148-4-200802190-00008

[B18] Sorensen J Davidsen M Gudex C Pedersen KM Bronnum-Hansen H Danish EQ-5D population norms Scand J Public Health 2009 37 5 467 474 10.1177/1403494809105286 19535407

[B19] Crossley K Bennell K Green S Cowan S McConnell J Physical therapy for patellofemoral pain: a randomized, double-blinded, placebo-controlled trial AmJSports Med 2002 30 6 857 865 10.1177/03635465020300061701 12435653

[B20] Crossley KM Vicenzino B Pandy MG Schache AG Hinman RS Targeted physiotherapy for patellofemoral joint osteoarthritis: a protocol for a randomised, single-blind controlled trial Bmc Musculoskel Dis 2008 9 122 10.1186/1471-2474-9-122 PMC2556332 18793446

[B21] Lesher JD Sutlive TG Miller GA Chine NJ Garber MB Wainner RS Development of a clinical prediction rule for classifying patients with patellofemoral pain syndrome who respond to patellar taping J Orthop Sports Phys Ther 2006 36 11 854 866 17154139 10.2519/jospt.2006.2208

[B22] Roos EM Roos HP Lohmander LS Ekdahl C Beynnon BD Knee Injury and Osteoarthritis Outcome Score (KOOS)--development of a self-administered outcome measure J Orthop Sports Phys Ther 1998 28 2 88 96 9699158 10.2519/jospt.1998.28.2.88

[B23] Rabin R de Charro F EQ-5D: a measure of health status from the EuroQol Group Ann Med 2001 33 5 337 343 10.3109/07853890109002087 11491192

[B24] Cowan SM Bennell KL Crossley KM Hodges PW McConnell J Physical therapy alters recruitment of the vasti in patellofemoral pain syndrome Med Sci Sport Exer 2002 34 12 1879 1885 10.1097/00005768-200212000-00004 12471291

[B25] Kelln BM McKeon PO Gontkof LM Hertel J Hand-held dynamometry: reliability of lower extremity muscle testing in healthy, physically active, young adults J Sport Rehabil 2008 17 2 160 170 18515915 10.1123/jsr.17.2.160

[B26] Rathleff MS Samani A Olesen CG Kersting UG Madeleine P Inverse relationship between the complexity of midfoot kinematics and muscle activation in patients with medial tibial stress syndrome J Electromyogr Kinesiol 2011 10.1016/j.jelekin.2011.03.001 21474335

[B27] Clark DI Downing N Mitchell J Coulson L Syzpryt EP Doherty M Physiotherapy for anterior knee pain: a randomised controlled trial Ann Rheum Dis 2000 59 9 700 704 10.1136/ard.59.9.700 10976083 PMC1753277

[B28] Richman JS Moorman JR Physiological time-series analysis using approximate entropy and sample entropy AmJPhysiol Heart CircPhysiol 2000 278 6 H2039 H2049 10.1152/ajpheart.2000.278.6.H2039 10843903

[B29] Cowan SM Bennell KL Hodges PW The test-retest reliability of the onset of concentric and eccentric vastus medialis obliquus and vastus lateralis electromyographic activity in a stair stepping task Physical Therapy in Sport 2000 1 4 129 136 10.1054/ptsp.2000.0036

[B30] Collins N Crossley K Beller E Darnell R McPoil T Vicenzino B Foot orthoses and physiotherapy in the treatment of patellofemoral pain syndrome: randomised clinical trial Br J Sports Med 2009 43 3 169 171 19270165 10.1136/bmj.a1735

[B31] Cowan SM Bennell KL Hodges PW Crossley KM McConnell J Delayed onset of electromyographic activity of vastus medialis obliquus relative to vastus lateralis in subjects with patellofemoral pain syndrome Arch Phys Med Rehab 2001 82 2 183 189 10.1053/apmr.2001.19022 11239308

[B32] Powers CM Perry J Hsu A Hislop HJ Are patellofemoral pain and quadriceps femoris muscle torque associated with locomotor function? Phys Ther 1997 77 10 1063 1075 discussion 1075-1068 9327821 10.1093/ptj/77.10.1063

[B33] Kaya D Citaker S Kerimoglu U Atay OA Nyland J Callaghan M Yakut Y Yuksel I Doral MN Women with patellofemoral pain syndrome have quadriceps femoris volume and strength deficiency Knee Surg Sports Traumatol Arthrosc 2011 19 2 242 247 10.1007/s00167-010-1290-2 20953760

[B34] Kettunen JA Harilainen A Sandelin J Schlenzka D Hietaniemi K Seitsalo S Malmivaara A Kujala UM Knee arthroscopy and exercise versus exercise only for chronic patellofemoral pain syndrome: 5-year follow-up Br J Sports Med 2011 10.1136/bjsm.2010.079020 21357578

[B35] Roos EM Lohmander LS The Knee injury and Osteoarthritis Outcome Score (KOOS): from joint injury to osteoarthritis Health Qual Life Outcomes 2003 1 64 10.1186/1477-7525-1-64 14613558 PMC280702

